# Incomplete Unilateral Horner’s Syndrome Due to Superior Vena Cava Compression Syndrome Caused by Small Cell Lung Carcinoma

**DOI:** 10.7759/cureus.68662

**Published:** 2024-09-04

**Authors:** Josef Finsterer

**Affiliations:** 1 Neurology, Neurology and Neurophysiology Center, Vienna, AUT

**Keywords:** horner’s syndrome, small cell lung carcinoma, ptosis, vena cava compression syndrome, myasthenic syndrome

## Abstract

Incomplete unilateral Horner's syndrome due to central small cell lung cancer (SCLC) with consecutive compression of the superior vena cava has not been reported before. A 56-year-old woman with stage T4,N3(cerv),M1a metastatic central SCLC treated with carboplatin and etoposide developed incomplete Horner's syndrome before receiving the first cycle of chemotherapy. Investigation for ptosis ruled out myasthenic syndrome, myasthenia, primary myopathy, facial palsy, and mitochondrial disorders. After congestion developed in the upper inflow area and compression of the superior vena cava was noted, Horner's syndrome was attributed to superior vena cava compression syndrome (SVCCS). Stenting of the stenosis did not result in a complete resolution of Horner's syndrome.

In summary, SVCCS can lead to congestion of the jugular veins and subsequent impairment of the centripetal sympathetic fibers that run along the carotid artery. Compression of the sympathetic fibers can lead to incomplete Horner's syndrome with non-fluctuating and non-exercise-induced ptosis. Clinicians should be aware that Horner's syndrome associated with SCLC may be due not only to a myasthenic syndrome but also, in rare cases, to a focal affection of sympathetic fibers.

## Introduction

Horner's syndrome is clinically characterized by ptosis, miosis, and enophthalmus and is most commonly due to carotid artery dissection, Pancoast tumor, thyroid malignancy, stellate ganglion blockage, cervical syringomelia, intrathoracic goiter, Wallenberg syndrome, neuroblastoma or cervical spine trauma [1]. Ptosis is explained by the denervation of the tarsalis muscle by sympathetic fibers, miosis by sympathetic denervation, and compensatory parasympathetic overactivity leading to pupillary constriction [1]. Unilateral Horner's syndrome due to upper inflow congestion caused by superior vena cava compression syndrome (SVCCS) has been rarely reported [2,3]. The pathophysiology of how SVCCS causes Horner's syndrome is explained by increased intravenous pressure leading to congestion of the jugular vein and superior vena cava and subsequent irritation of sympathetic fibers running along the carotid artery to the brain. To our knowledge, incomplete, unilateral Horner's syndrome due to central small cell lung cancer (SCLC) with consecutive compression of the superior vena cava has not yet been reported.

## Case presentation

**Figure 1:**
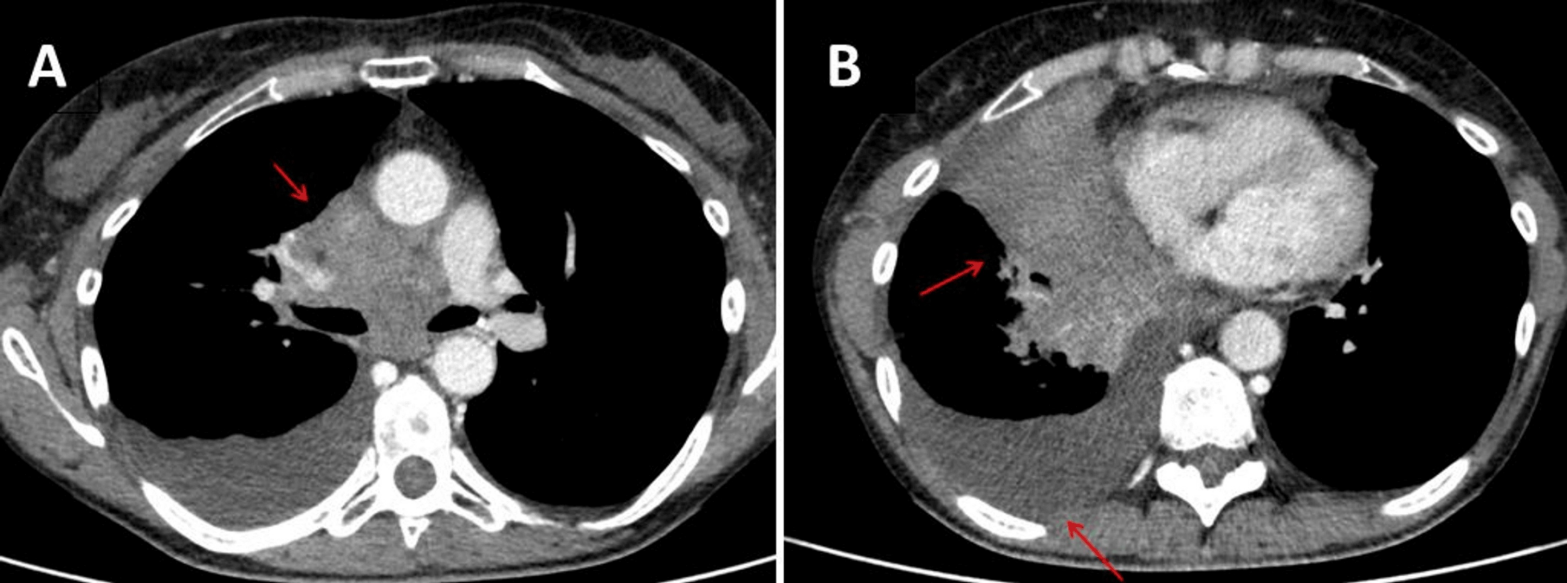
CT scan of the chest showing a central mass compressing the superior vena cava (A). Tumor growth was also complicated by pleural effusion and atelectasis of the middle lobe bronchus (B).

**Figure 2:**
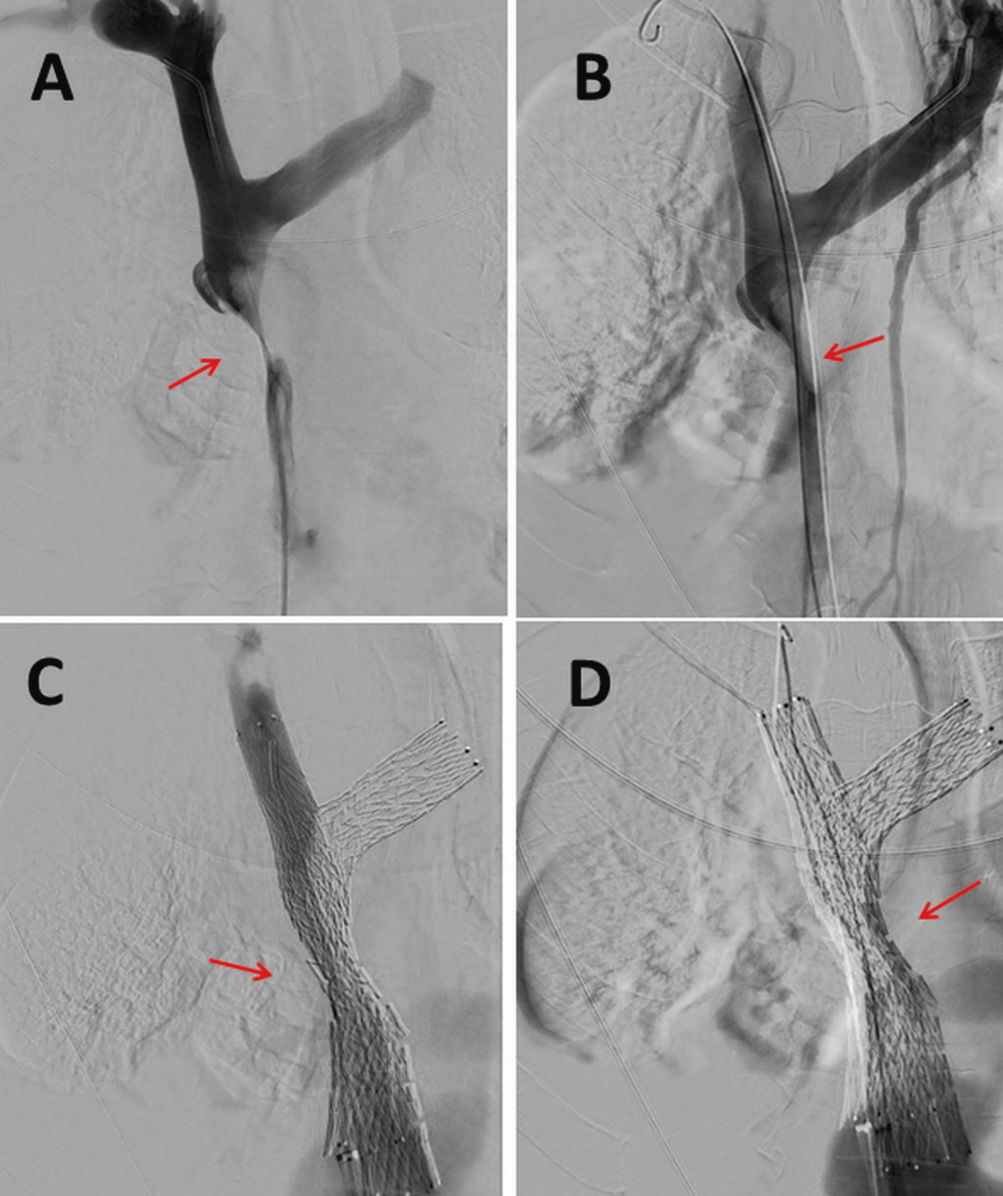
The cavography shows a stenosis of the superior vena cava (A), which could be passed through and enlarged with catheters (B) and could be successfully stented using balloon angioplasty (C), so that the inflow blockage resolved, but the Horner’s syndrome only marginally improved (D).

The patient is a 56-year-old woman with SCLC metastatizing in the left perirenal area, left adrenal gland, and right cervical lymph nodes. The SCLC was first diagnosed three months earlier on a biopsy of cervical lymph nodes, categorized as T4, N3, M1a and treated with carboplatin and etoposide. The tumor also caused compression of the superior vena cava, resulting in SVCCS with congestion of the upper inflow, which is why the vena cava had to be stented (Figures 1A, 1B). In addition, rivaroxaban was administered for anticoagulation to prevent thrombosis. The tumor growth also led to compression of the right middle lobe bronchus with consecutive atelectasis, pneumonia, and pleural effusion (Figures 2A-2D), which required repeated pleural punctures. Even before the first chemotherapy, she had noticed unilateral ptosis of the right upper eyelid, which did not fluctuate with the time of day and did not increase with exertion. Her medical history also included cervical syndrome, migraine with aura since childhood, nicotine abuse until one year before the discovery of SCLC, and asthma.

On admission for the third cycle of chemotherapy, the clinical neurological examination revealed ptosis of the right upper eyelid and miosis on the right, but no enophthalmus, no bulbar deficiency, and no diplopia (incomplete Horner’s syndrome). The Simpson and ice tests were negative. The pupillary reaction in the right eye was weak. There was discrete weakness (M5-) of the right upper limb and subclonic Achilles tendon reflexes, but the rest of the neurologic examination was normal. Blood work revealed anemia, leukocytosis, thrombocytosis, elevated CRP, and elevated levels of GOT, GGT, ALP, and LDH. Neuron-specific enolase (NSE) was elevated to 33.6 µg/L (n, 0-16.3 µg/L), but CYFRA was normal. No abnormal increment or decrement was observed with high and low-frequency repetitive nerve stimulation. Antibodies against the postsynaptic acetylcholine receptor and MUSK as well as against the presynaptic voltage-gated calcium channels (VGCC) were negative. The patient received iron and blood transfusions due to anemia (Table 1). For pain, she received metamizol, tetra-hydrocannabinol, and hydromorphone. Further tumor growth could be prevented by chemotherapy. The ptosis did not completely resolve by the time of discharge. Her medications at discharge included pantoprazole, ondansetron, iron, beclomethasone/formoterol, metamizole, dronabinol, and hydromorphone.

**Table 1:** Blood test results obtained during hospitalization CRP: C-reactive protein, HD: hospital day, RL: reference limits, GOT: glutamic oxaloacetic transaminase, GPT: glutamic pyruvic transaminase, GGT: gamma-glutamyltransferase, ALP: alkaline phosphatase

Parameter	HD5	HD24	HD45	RL
Erythrocytes	3.5	3.2	3.0	4.0-5.0 T/L
Hemoglobin	11.0	9.9	9.1	13.0-16.0 g/dL
Hematocrit	30.5	28.5	27.1	38.0-45.0%
Leukocytes	20.6	12.1	30.1	4.0-10-0 G/L
Thrombocytes	537	1158	768	150-400 G/L
CRP	6.4	219.5	88.2	0.0-4.9 mg/L
GOT	22	39	56	0-34 U/L
GPT	6	9	16	0-34 U/L
GGT	22	167	190	0-37 U/L
LDH	401	805	967	0-246 U/L
ALP	79	233	198	30-120 U/L

## Discussion

The presented patient is interesting because of unilateral ptosis and miosis in the context of an incomplete Horner's syndrome caused by SVCCS. The SVCCS resulted from an expanding, metastatic central SCLC after many years of nicotine abuse. Horner's syndrome was classified as incomplete as the patient had no enophthalmus. Although unilateral ptosis has been previously reported in association with SCLC [4], it is usually due to Lambert-Eaton myasthenic syndrome (LEMS) [4,5]. LEMS is clinically characterized by proximal muscle weakness that usually begins in the legs and can spread to the bulbar and extraocular muscles and, in severe cases, to the respiratory muscles with respiratory insufficiency [6]. In the index patient, no muscles other than the tarsalis muscle were affected. LEMS is caused by antibodies directed against presynaptic P/Q-type VGCCs localized in motor nerve endings and the autonomic nervous system (ANS) [6]. These antibodies are found in 90% of patients with LEMS [6]. Since LEMS is associated with SCLC in 50%-60% of cases [6], it is important to diagnose it early and initiate appropriate symptomatic and immunosuppressive treatment [6].

After the exclusion of LEMS by high-frequency repetitive nerve stimulation and negativity of VGCC antibodies, central or peripheral facial palsy and primary myopathy, in particular mitochondrial myopathy, were considered alternative causes for the ptosis in the index patient. However, cerebral MRI, viral panels, and CSF examinations were inconclusive. Family history was also negative for a primary myopathy involving the eye muscles, and there was no clinical evidence of a mitochondrial disorder, particularly chronic progressive external ophthalmoplegia (CPEO), Kearns-Sayre syndrome (KSS), or myo-neuro-gastrointestinal encephalopathy (MNGIE).

After the exclusion of LEMS and myasthenia, primary myopathy, and mitochondrial disorder, Horner's syndrome was attributed to SVCCS. SVCCS is caused by compression, invasion, or thrombosis of the superior vena cava and/or brachiocephalic veins [7]. SVCCS can be caused by a benign tumor or malignancy [7]. The clinical manifestations of SVCCS range from asymptomatic cases to rare life-threatening emergencies with upper airway obstruction and increased intracranial pressure [7]. Increased venous pressure in SVCCS can even lead to intracranial venous pressure [2]. When the intracranial venous pressure caused by SVCCS is high, the clinical picture may resemble a cavernous-dural arteriovenous fistula [2]. The symptoms of SVCCS usually correlate with the severity and extent of venous obstruction and inversely with the development of venous collateral circulation [7]. SVCCS complicated by Horner's syndrome has previously been described in a patient with intrathoracic goiter [8]. Incomplete unilateral Horner's syndrome due to SVCCS has also been reported in a patient with fibrosing mediastinitis [9]. In addition, Horner's syndrome due to SVCCS has been reported in a 51-year-old woman and a 41-year-old man with mediastinal neurolemmoma [10,11], in a young woman with mediastinal hydatid cyst [12] and a 67-year-old woman with mediastinal hydatid cyst [13].

One argument against SVCCS as a cause of ptosis in the index case is that the ptosis did not completely disappear after cava stenting. Resolution of the neck congestion should also have resulted in a reduction in pressure on the sympathetic fibers. Whether the enlarged cervical lymph nodes contributed to the compression of the sympathetic fibers along the right carotid artery remains speculative, but since they were described as being located supraclavicularly, it is unlikely that they affected the sympathetic fibers.

## Conclusions

This case demonstrates that SVCCS can lead to congestion of the jugular veins and subsequent impairment of the centripetal sympathetic fibers that run along the carotid artery. Compression of sympathetic fibers can lead to incomplete Horner’s syndrome with non-fluctuating and exercise-independent ptosis. Horner’s syndrome can persist as long as chemotherapy has not resulted in a significant reduction in tumor mass. Clinicians should be aware that the ptosis associated with SCLC may be due not only to LEMS, but in rare cases also to focal impairment of sympathetic fibers, clinically manifesting as incomplete Horner’s syndrome.
